# Plasma-derived extracellular vesicles yield predictive markers of cranial irradiation exposure in mice

**DOI:** 10.1038/s41598-019-45970-x

**Published:** 2019-07-01

**Authors:** Charles P. Hinzman, Janet E. Baulch, Khyati Y. Mehta, Michael Girgis, Shivani Bansal, Kirandeep Gill, Yaoxiang Li, Charles L. Limoli, Amrita K. Cheema

**Affiliations:** 1 Department of Biochemistry, Molecular and Cellular Biology, Georgetown University, Georgetown University Medical Center, Washington, D.C 20057 USA; 2 Department of Radiation Oncology, University of California, Irvine, CA 92697 USA; 3 Lombardi Comprehensive Cancer Center, Department of Oncology, Georgetown University Medical Center, Washington, D.C 20057 USA

**Keywords:** CNS cancer, Prognostic markers, CNS cancer

## Abstract

Ionizing radiation exposure to the brain is common for patients with a variety of CNS related malignancies. This exposure is known to induce structural and functional alterations to the brain, impacting dendritic complexity, spine density and inflammation. Over time, these changes are associated with cognitive decline. However, many of these impacts are only observable long after irradiation. Extracellular vesicles (EVs) are shed from cells in nearly all known tissues, with roles in many disease pathologies. EVs are becoming an important target for identifying circulating biomarkers. The aim of this study is to identify minimally invasive biomarkers of ionizing radiation damage to the CNS that are predictors of late responses that manifest as persistent cognitive impairments. Using a clinically relevant 9 Gy irradiation paradigm, we exposed mice to cranial (head only) irradiation. Using metabolomic and lipidomic profiling, we analyzed their plasma and plasma-derived EVs two days and two weeks post-exposure to detect systemic signs of damage. We identified significant changes associated with inflammation in EVs. Whole-plasma profiling provided further evidence of systemic injury. These studies are the first to demonstrate that profiling of plasma-derived EVs may be used to study clinically relevant markers of ionizing radiation toxicities to the brain.

## Introduction

Cognitive impairments due to radiation treatment for cancers of the central nervous system (CNS) are well documented^1,2^. However, the resultant structural and functional changes leading to cognitive decline tend to appear well after exposure to radiation therapy^3,4^. Given that this cognitive decline is a delayed normal tissue response, early noninvasive biomarkers of adverse late outcomes may reveal therapeutic strategies to ameliorate the effects of clinical radiotherapy and improve the quality of life for patients. Biomarkers specific to an irradiated target organ have proven elusive, confounded by the background of the circulating secretome derived from the myriad of exposed cell types. In particular, this has and remains a challenge for the CNS, given the marked latency of functional outcomes and the disconnect between early and late effects in such late responding organs^5^. Early changes in inflammation and oxidative stress follow cyclical patterns over protracted irradiation intervals, complicating predictive assessments of underlying biology, including efforts to identify circulating biomarkers specific to target tissues and constitutive cell types. Therefore, to resolve this knowledge gap between early and late events in the irradiated CNS, we undertook a metabolomics approach of circulating extracellular vesicles (EVs) to elucidate possible biomarkers of CNS radiation exposure.

EVs are nanometer sized particles released from cells in every tissue type^6,7^. EVs are characterized as either exosomes or microvesicles. Exosomes are defined as lipid membrane containing EVs with a diameter of less than 150 nm, originating from intracellular multivesicular bodies^6,8^. Microvesicles tend to be larger (50–1000 nm) and originate directly from budding at the plasma membrane^9^. EVs contain a variety of cargo, including proteins, nucleic acids, lipids and other bioactive molecules^6^, and can be isolated from cell culture, plasma and tissue. EVs are known to play important roles in mediating cell-to-cell communication in normal physiology as well as in a variety of pathologies^10–12^. While many researchers are studying the diagnostic and therapeutic capabilities of EVs^13–20^, their role in the context of ionizing radiation exposure remain unclear. Previous studies have shown that EV secretion is increased with ionizing radiation exposure in a time and dose dependent manner^21,22^. Others have described a potential role of EVs in the bystander effect during cancer treatment^23–26^. However, the systemic metabolomic and/or lipidomic profiles of EVs in mammals exposed to cranial ionizing radiation have not yet been studied.

To identify early molecular markers that are predictive of late radiation induced cognitive changes, we examined the plasma and plasma-derived EVs from mice exposed to 9 Gy cranial radiation. 9 Gy was chosen to emulate the partial radiotherapy paradigm used for glioma patients. Standard radiation dosing for these patients utilizes a 60 Gy total dose, delivered in 2 Gy fractions leading to a biologically effective dose (BED) of 100 Gy (α/β = 3 for brain)^27,28^. Other research groups which have implemented altered fractionation schedules target this same BED value to elicit normal tissue damage in brain studies analyzing late CNS effects^27–30^. Thus, for these studies we have selected only one-third of this fractionation, 8.65 Gy (rounded to 9 Gy).

We have recently reported on increased endoplasmic reticulum (ER) stress in the hippocampal tissue of this same cohort of mice, as early as two days post-exposure^31^. In this follow-up study, we employed a combination of UPLC-MS, UPLC-MS/MS and GC-MS to characterize the metabolomic and lipidomic profiles of plasma and plasma-derived EVs in these mice. Profiling of plasma-derived EVs identified significant enrichment of markers indicating a systemic inflammatory response including triglycerides, platelet activating factor, carnitine and C-16 sphinganine. Additionally, we identified significant down-regulation of palmitic amide in EVs. These metabolites were not found to be significantly dysregulated in whole plasma profiling, suggesting they may be EV-cargo specific. Importantly, whole plasma profiling yielded complementary information where we observed significant systemic down-regulation of β-hydroxybutyric acid, a neuroprotectant^32,33^ and anti-inflammatory molecule^34^. Taken together, these results demonstrate the utility of profiling EVs from peripheral blood in the context of cranial irradiation for early identification of normal tissue injury.

## Results

### Plasma-derived EVs characterization and quantification

We began our investigation by isolating EVs from the plasma of mice cranially irradiated using a dose of 9 Gy x-rays. Characterization of the EV fraction was accomplished using nanoparticle tracking analysis (NTA) and quantitative ELISA. Initial concentrations were estimated using a CD63 ELISA (Supplementary Fig. S1). We identified a drastic increase in the concentration of systemic EVs derived from irradiated mice two days post-irradiation (Supplementary Fig. S1). Previous reports have shown increased EV shedding post-irradiation. However, our concentrations according to NTA did not recapitulate this increase (Supplementary Table S1). Since our two characterization methods did not identify the same difference, it remains important to characterize EV samples using multiple modalities. In terms of the size of vesicles isolated, NTA revealed similar mean diameters for our EV samples (Supplementary Table S1). Interestingly, the mode diameter size was considerably different between sham and irradiated groups, though statistically significant only at the 2-week time point (Supplementary Fig. S1); however, it remains unclear if this is biologically relevant.

### EVs carry markers of inflammation post-cranial irradiation

**Figure 1:**
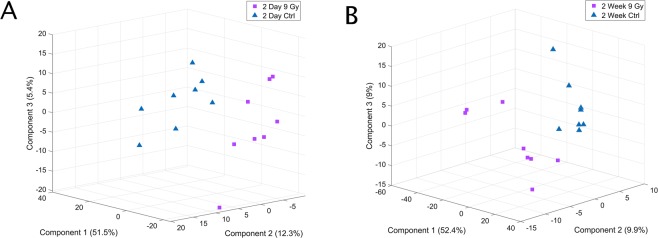
EV biomarker profiles can separate irradiated mice from controls. Partial least squares-discriminant analysis (PLS-DA) demonstrating separation between 9 Gy cranially irradiated mice and sham irradiated mice (**A**) 2 days or (**B**) 2 weeks post-irradiation.

To assess the potential of EVs as informative biomarkers of cranial irradiation, we performed untargeted metabolomics profiling using UPLC-QToF-MS. Initial examination of the total features captured, using partial least squares-discriminant analysis (PLS-DA), indicated separation between the molecular profiles of EVs from irradiated mice compared to sham-irradiated mice (Fig. 1). Somewhat surprisingly, EV profiles revealed a complete separation of sham and irradiated groups suggesting a robust systemic molecular response even though the radiation exposure was limited to the head. Using our well validated metabolomic and lipidomic profiling platform, we identified ~2,100 *m/z*’s that were significantly altered in EVs from irradiated mice compared to sham-irradiated mice. Ultimately, we putatively annotated 24 metabolites that were significantly changed in EVs (Supplementary Table S2).

**Figure 2:**
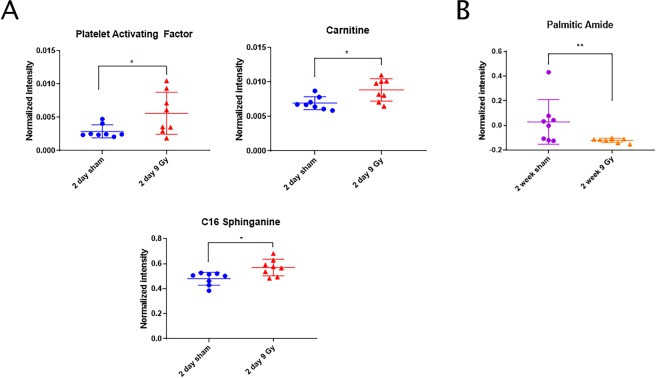
EVs can be profiled for markers of inflammation post-cranial irradiation. Box plots for key metabolites implicated in an inflammatory response (**A**) 2 days or (**B**) 2 weeks after 9 Gy cranial radiation exposures. P-values: *≤0.05, **≤0.01.

**Figure 3:**
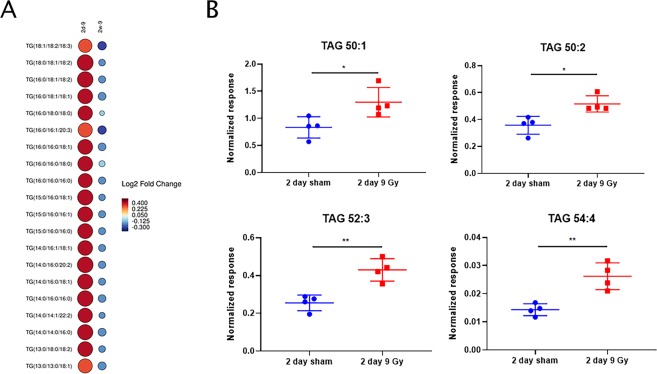
Triglycerides (TGs) accumulate in EVs post-cranial irradiation. (**A**) Modified heat map showing significant enrichment of various TG species 2 days or 2 weeks post-irradiation. Color corresponds to log2 fold change with red indicating up-regulation and blue indicating down-regulation, compared to sham irradiated mice. The size of each circle is the –log10 of each P-value, with a larger circle indicating more significant P-value than smaller circles. (**B**) Targeted MRM-MS quantification of TG’s in 2 day sham and 2 day 9 Gy irradiated mouse EV samples. Up-regulation is observed for each TG, validating our untargeted findings. P-values: *≤0.05, **≤0.01.

We observed the enrichment of several molecules in EVs that are known to be associated with inflammation 2 days’ post-irradiation, including C16 sphinganine, carnitine and platelet activating factor (Fig. 2A). We also identified a significant decrease in palmitic amide at 2 weeks post-irradiation (Fig. 2B). Additionally, we observed enrichment of several triglyceride (TG) species in EVs 2 days post-irradiation (Fig. 3A, Table 1). TG levels eventually decreased to levels below their sham-irradiated matched mice 2 weeks post-irradiation, though these differences were not statistically significant (Fig. 3A). These are striking findings given that none of these compounds were significantly changed in total plasma profiling, suggesting they are EV-specific and functionally relevant. Lipid accumulation has previously been associated with whole-body ionizing radiation exposure^35^, and dyslipidemia is an outcome we have observed in other radiation studies^22^.

**Table 1:** Putatively annotated triglyceride (TG) species up-regulated 2 days post 9 Gy cranial irradiation.

m/z	RT	Name	Fold Change	FDR adjusted p-value	CID Fragments
766.6917	7.37	TG(13:0/13:0/18:1)	1.26	4.997E-03	467.4113
768.7066	7.55	TG(14:0/14:0/16:0)	1.29	4.101E-03	523.4706, 495.4398
808.7384	7.67	TG(15:0/16:0/16:1)	1.29	4.164E-03	549.4877, 537.4888
810.7536	7.83	TG(15:0/16:0/16:0)	1.29	4.101E-03	551.5031, 537.4881
820.739	7.62	TG(14:0/16:1/18:1)	1.28	4.201E-03	575.5038, 549.4877, 521.4562
824.7693	9.44	TG(16:0/16:0/16:0)	1.28	4.306E-03	551.5026
829.7246	7.92	TG(14:0/14:1/22:2)	1.32	4.659E-03	829.7254, 603.5342, 601.5186
834.755	7.71	TG(13:0/18:0/18:2)	1.28	4.164E-03	603.5304, 537.4888
836.7696	7.85	TG(15:0/16:0/18:1)	1.31	4.101E-03	577.5176, 563.504, 537.4881
848.7703	7.8	TG(14:0/16:0/20:2)	1.3	4.101E-03	848.7692, 603.5324, 575.5029, 523.4729
850.7847	7.93	TG(16:0/16:0/18:1)	1.3	4.192E-03	850.7848, 577.5183, 551.5026
852.8002	8.09	TG(16:0/16:0/18:0)	1.32	4.164E-03	852.8014, 579.5351, 551.5042
872.7701	7.64	TG(16:0/16:1/20:3)	1.27	4.981E-03	601.5196, 599.5042, 549.4877
874.7849	7.79	TG(16:0/18:1/18:2)	1.3	4.101E-03	601.5183, 577.5176, 575.5029
876.8004	7.94	TG(16:0/18:1/18:1)	1.29	4.128E-03	603.5342, 577.5183
880.8318	8.26	TG(16:0/18:0/18:0)	1.32	4.627E-03	607.5655, 579.5358
896.7699	7.48	TG(18:1/18:2/18:3)	1.27	7.116E-03	896.7708, 601.5156, 599.5037, 597.4888
907.773	7.96	TG(18:0/18:1/18:2)	1.33	4.101E-03	605.5452, 603.5342, 601.5186

Given the functional relevance of TG accumulation, we performed targeted UPLC-MS/MS to validate and quantify these putative annotations. Using a validated MRM method^36^ we attempted to quantify 10 known species of TGs in a subset of randomly chosen EV samples (*n* = 4 per group). Of these, we were able to obtain reproducible spectra for 7 TGs in our EV samples with a signal to noise (S/N) > 10 for reliable quantification, with 4 TGs being significantly up-regulated in the irradiated group 2 days post-irradiation, validating our untargeted findings (Fig. 3B).

Significantly, EV TG profiles were able to discriminate mice that were cranially irradiated from those which were sham irradiated. Employing a receiver operating characteristic (ROC) curve, we evaluated the efficacy of a TG biomarker panel using the putatively annotated TG species. We found that even with only 2 TG species [TG(14:0/16:0/20:2) and TG(16:0/18:1/18:2)] it was possible to discriminate between groups (Supplementary Fig. S2) with >90% specificity and sensitivity. Importantly, the TG species that were accurately quantified in our LC-MS/MS methods mirrored this ability (Supplementary Fig. S3).

### Plasma profiling is synergistic with metabolomic profiles of EVs

Though our primary interest for this study was to understand the potential of EVs as biomarkers for damage due to cranial irradiation, we asked if profiling total plasma would yield overlapping and/or complementary information. We also sought to compare the increased TG levels in isolated EVs to TG levels in total plasma. Using our same targeted LC-MS/MS method, we quantified the same TG species we identified as up-regulated in EVs in plasma samples. We found that TG levels were only moderately increased in total plasma and this difference was not statistically significant (Supplementary Fig. S3).

**Figure 4:**
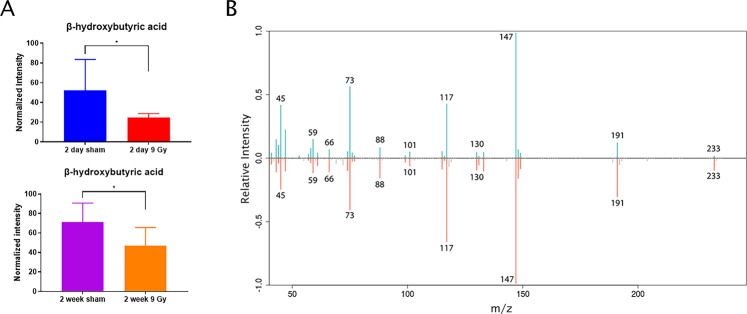
Systemic levels of β-hydroxybutyric acid are significantly decreased post-cranial irradiation. (**A**) Systemic levels of β-hydroxybutyric acid 2 days (top) or 2 weeks (bottom) post 9 Gy cranial irradiation. (**B**) Representative matching GC-MS spectra for β-hydroxybutyric acid detected in our samples (top) and from a pure chemical standard (bottom).

Importantly, global plasma metabolomic characterization showed radiation exposure induced robust changes in metabolite abundance. Using GC-MS, we analyzed the metabolite composition of our plasma samples and found a significant decrease in the levels of systemic β-Hydroxybutyrate (3-Hydroxybutyrate, BHB). This decrease was observed 2 days post-irradiation and persisted through the 2-week time point (Fig. 4). Initially thought of as a passive carrier of energy, BHB has proven to be an important signaling molecule in a variety of processes including neuroprotection and anti-inflammatory responses^34,37–39^.

## Discussion

Exposure to ionizing radiation is known to induce a complex cascade of molecular events that mediate the cellular response to radiation exposure. We have previously shown that cranial irradiation results in gross structural and functional changes in the brain that manifest over time. However, one of the outstanding questions in the field is whether partial body exposures lead to systemic alterations in metabolomic and lipidomic profiles and further if these changes can be leveraged as predictive biomarkers of radiation toxicities in the brain.

We used a quantitative and qualitative molecular phenotyping approach to delineate radiation-induced changes in the profiles of plasma and plasma-derived EVs. Our results demonstrate that EV profiling yields complementary information to traditional, total-plasma profiling for investigating biomarkers of cranial ionizing radiation exposure. NTA revealed potential differences in the morphology of EV populations post-irradiation. These differences should be further investigated. Additionally, concentration measurements were different between NTA and CD63 ELISA. We hypothesize that potentially, both measures may be accurate. It is possible to consider that CD63 signaling could be amplified in the context of radiation exposure, and for some reason EVs are overexpressing this protein.

EV metabolomic and lipidomic profiling revealed enrichment of a number of molecules involved in inflammation that may mediate systemic response to radiation exposure with potential impact on distant organ sites. For example, platelet activating factor was up-regulated 2 days post-irradiation. This lipid species has been associated with inflammation and intracranial endoplasmic reticulum (ER) stress signaling, a phenomenon we recently characterized in our hippocampus tissue analysis^31,40,41^. Furthermore, we observed a sharp increase in TGs 2 days post-irradiation that dissipated by 2 weeks. Previous studies have demonstrated that TGs increase in rat serum post-cranial irradiation^42^. However, this is the first time that TGs have been reported as being up-regulated in plasma-derived EVs post-cranial irradiation. Importantly, we did not see a sustained up-regulation of TG species in our plasma samples.

Additionally, palmitic amide is a primary fatty acid amide (PFAM), derived from the most abundant fatty acid, palmitic acid^43^. PFAM’s are incredibly important mediators of cell signaling in the mammalian nervous system^43^. The observed decrease in palmitic amide could be indicative of early-abrogated CNS signaling 2 weeks post-irradiation. Elevations in carnitine and sphinganine may reflect oxidative breakdown of lipid macromolecules from the irradiated CNS, possibly impacting neuroinflammation and mitochondrial energetics^44^. Lipids, myelin, and other fatty acids broken down intracellularly in the irradiated brain could be packaged into EVs and circulate to the periphery, contributing to the onset of radiation-induced neuroinflammation and degenerative pathways^45^. Further investigations are required to elucidate these mechanisms.

Our global plasma profiling revealed that TG levels were moderately increased, though not to statistical significance. This finding demonstrates the utility of probing EVs for biochemical profiles that may indicate biological changes missed by total plasma profiling. Importantly, we also identified a significant decrease in BHB, an outcome indicative of systemic stress. BHB, the most abundant ketone body in mammals, is synthesized in the liver from fatty acids^46^. Systemic BHB levels are highest during fasting and is one of the key results of ketosis and/or a ketogenic diet^47^. In fact, researchers are currently investigating the role of a ketogenic diet in the context of radiation response in malignant glioma and believe that BHB could potentiate the radiation response^47,48^.

When considering increased EV TGs in conjunction with decreased BHB and palmitic amide, it is possible that we have identified a brain-liver radioresponsive axis that is detectable weeks or months before cognitive impairment is observed. Traumatic brain injury has been associated with rapid changes in hepatic function including inflammation^49^. A recent study has shown that the brain undergoes a radiation response upon liver irradiation^50^. We hypothesize the reciprocal response can also be true, and our findings could indicate this phenomenon.

In total, we demonstrate the utility of probing and analyzing EVs for detailed information regarding cranial irradiation. Taken together, our identified markers could indicate early-onset damage that may lead to radiation-induced cognitive impairment.

## Methods

### Plasma collection and EV isolation

All animal procedures described in this study are in accordance with NIH guidelines and approved by the University of California Institutional Animal Care and Use Committee. C57BL/6J two-month old male mice were purchased from the Jackson Laboratory (Bar Harbor, ME). Details regarding animal housing, experimental design and irradiation protocols, were previously described^31^. Blood was collected at 2 days and 2 weeks after irradiation by cardiac puncture using syringes containing 0.5 M EDTA (~10% final blood volume). Following room temperature incubation, blood samples were centrifuged at 2,000 g at 4 °C to separate plasma. 100 mL of plasma was reserved and frozen at −80 °C for metabolomics analysis. The remainder was used for EV isolation as previously described, with minor modifications^51^. In short, plasma samples were thawed on ice and 200 µL of plasma was diluted in 1.5 mL of 1X PBS. Samples were centrifuged for 20 minutes at 16,000 × g, 4 °C to remove macromolecules and cell particulates. The supernatant was then transferred to sterile ultracentrifuge tubes, balanced with 1X PBS and ultracentrifuged at 100,000 × g, 4 °C for 2 hours. The supernatant was aspirated and EV pellets were re-suspended in 50 µL of 1X PBS and stored −80 °C until further processing. Plasma and EV samples were then shipped on dry ice to Georgetown University Medical Center for metabolomic and lipidomic analysis.

### Plasma and EV sample preparation for mass spectrometry

For plasma samples, 5 µL of plasma was added to 95 µL of chilled 40% isopropanol +25% methanol +35% water containing internal standards (debrisoquine and 4-nitrobenzoic acid). Samples were then vortexed and incubated on ice for 20 minutes. Next, 100 µL of pre-chilled acetonitrile was added to each sample. Samples were then vortexed and incubated at −20 °C for 15 minutes, followed by centrifugation at 13,000 rpm for 20 minutes at 4 °C. The supernatant from each sample was then transferred to mass spectrometry vials for data acquisition.

For the EV fraction, samples (EVs re-suspended in 1X PBS) were plunged in dry ice for 30 seconds, followed by heat shock in a 37 °C water bath for 90 seconds. This was repeated 2 more times. Samples were then sonicated for 30 seconds, vortexed and incubated on ice for 20 minutes. Next, 150 µL of chilled 40% isopropanol +25% methanol +35% water containing internal standards (debrisoquine and 4-nitrobenzoic acid) and 150 µL of chilled acetonitrile were added to samples. Samples were then vortexed and incubated at −20 °C for 30 minutes. Finally, samples were centrifuged at 4 °C, 13,000 rpm for 20 minutes and supernatant was transferred to mass spectrometry vials for data acquisition. Protein pellets were stored at −80 °C for subsequent protein quantification.

### EV characterization using quantitative ELISA and nanoparticle tracking analysis

Characterization of EV enrichment was accomplished using quantitative ELISA and nanoparticle tracking analysis (NTA). EV concentration was determined using an ExoELISA-ULTRA Complete CD 63 detection kit (System Biosciences, Palo Alto, CA), in accordance with manufacturer’s protocol. This ELISA detects CD63, a well-established membrane marker for EVs^52–55^. Total protein concentration was obtained via Bradford assay. NTA was accomplished using a NanoSight NS300 (Malvern Panalytical, Malvern, UK) equipped with a high sensitivity sCMOS camera, 532 nm laser and automatic syringe pump. EV samples were diluted 1:10 with HPLC-grade water. Videos were captured and processed using NTA 3.3 Dev Build 3.3.104 (Malvern) with 3 videos of 60 s per measurement. A total of 1500 frames were examined per sample. EV sample characteristics, as well as camera level and detection settings, are listed in Supplementary Table S1.

### UPLC-MS untargeted metabolomics and lipidomics

Metabolomic and lipidomic data were acquired as previously reported^31^. Raw data were pre-processed in R using XCMS (Scripps Institute, La Jolla, CA). For plasma samples, intensity values were normalized to intensities of internal standards. For EV samples, intensity values were normalized to intensities of internal standards and total protein concentration.

### UPLC-MS/MS triglyceride (TG) quantification

The LC and MRM methods for quantification of TGs used here were developed by Waters corporation^36^. Briefly, EV samples (20 µL) were mixed with 50 µL of isopropanol: methanol: water (35:25:40) containing an internal standard (PC: 16:0-d31-18:1 10 ng/mL). Plasma samples (25 µL) were mixed with 100 µL of isopropanol containing an internal standard (PC: 16:0-d31-18:1 50 ng/mL). Samples were vortexed and incubated on ice for 20 minutes. The samples were incubated at −20 °C for 20 minutes. Finally, samples were centrifuged at 13,000 rpm at 4 °C for 10 minutes; supernatant was then transferred to MS vials for analysis. Targeted quantitation of triglycerides (TAG 50:1, TAG 50:2, TAG 52:3, TAG 54:4) was performed using multiple reaction monitoring (MRM) mass spectrometry. 5 µL of EV samples or 2 µL of plasma samples were resolved on a CORTECS T3 2.7 µm, 2.1 × 30 mm column online with a triple quadrupole mass spectrometer (Xevo-TQ-S, Waters Corporation, Milford, MA). Signal intensities from all MRM Q1/Q3 ion pairs for the triglycerides were ranked to ensure selection of the most intense precursor and fragment ion pair for MRM-based quantitation. This approach resulted in selection of cone voltages and collision energies that maximized the generation of each fragment ion species; MRM parameters are specified in Supplementary Table S3. The metabolite ratios were calculated by normalizing the peak area of endogenous metabolites within EV samples, normalized to the internal standard. The sample queue was randomized, and solvent blanks were injected to assess sample carryover using four biological replicates for each comparative group. Data pre-processing was performed using TargetLynx v3.0 software (Waters Corporation).

### GC-MS sample preparation and derivatization

25 µL of plasma was added to ice cold 250 µL methanol containing an internal standard (4-nitrobenzoic acid) and mixed for 2 minutes. Samples were then centrifuged at 4 °C, 13,000 rpm for 20 minutes. The supernatant was separated, and the residue was further extracted with 250 µL ice cold methanol. Next, 250 µL 1 M KOH solution in methanol was added to each sample and samples were mixed for 30 minutes. Sample pH was stabilized to 5 with HCl in distilled water and 1 mL of isooctane was then added to each sample and mixed for 5 minutes. Finally, samples were centrifuged for 20 minutes at 4 °C, 13,000 rpm. The isooctane supernatant layer was then combined with the methanol extracts from the previous steps. Samples were placed in GC vials and evaporated using a speedvac.

Derivatization was accomplished by adding 20 µL of methoxyamine (20 mg/mL) to the dried samples and heating in an agitator at 60 °C for 30 minutes. Subsequently, 100 µL of N-Methyl-N-(trimethylsilyl) trifluoroacetamide (MSTFA) was added. Vials were then again placed into an agitator at 60 °C for 30 minutes. Finally, vials were capped and data was acquired.

### GC-MS profiling

1.5 µL of each derivatized sample was injected in (1:5) split mode into an Agilent 7890 B GC system (Agilent Technologies, Santa Clara, CA) coupled to a Pegasus HT TOF-MS (LECO Corporation, St. Joseph, MI). Separation was achieved on an Rtx-5 w/Integra-Guard capillary column (30 m × 0.25 mm ID, 0.25 µm film thickness; Restek Corporation, Bellefonte, PA) with helium as the carrier gas, at a constant flow rate of 1.0 mL/minutes. The temperatures of injection, transfer interface and ion source were 150 °C, 270 °C and 320 °C, respectively. GC temperature programming was set to 0.2 minutes of isothermal heating at 70 °C, followed by 6 °C /minutes oven temperature, ramping to 300 °C, a 4.0 minute isothermal heating of 270 °C, 20 °C/minute to 320 °C and a 2.0 minute isothermal heating of 320 °C. Electron impact ionization (70 eV) at full scan mode (40–600 *m/z*) was used, with an acquisition rate of 20 spectra per second in the TOF/MS setting.

Peak picking and alignments were performed using ChromaTof 4.7.2 (LECO Corporation). Mass spectra were compared to literature spectra available in the NIST database as well as the Fiehn library of compounds. A β-hydroxybutyric acid pure chemical standard was purchased (catalog #166898, Sigma-Aldrich, St. Louis, MO) for validation.

### Statistical analysis

Multivariate statistics were performed using Metaboanalyst V3.0 (Xia lab, McGill University, Montreal) and custom R scripts, with log transformation and Pareto scaling. All reported comparisons were binary comparisons using Student’s two-tailed t-tests with homogenous variance. *m/z*’s with FDR-adjusted P < 0.05 were considered significant and annotated using tandem mass spectrometry and SIMLIPID software V6.03 (Premier Biosoft, Palo Alto, CA), or with pure chemical standards as described. Figures were generated with Metaboanalyst V3.0, GraphPad Prism 7 (GraphPad Software, La Jolla, CA), MATLAB version R2019a (MathWorks, Natick, MA) and custom R scripts.

## Supplementary information

Supplemental Figures S1-S3

Supplementary Tables

## Data Availability

The datasets generated and/or analyzed during the current study are available from the corresponding author on reasonable request.
