# Supplementary material for: Plasma-derived extracellular vesicles yield predictive markers of cranial irradiation exposure in mice
Source: Sci Rep. 2019 Jul 1;9:9460. doi: 10.1038/s41598-019-45970-x (PMC6603161; doi:10.1038/s41598-019-45970-x)

Supplementary Figures for:

Plasma-derived extracellular vesicles yield predictive markers of cranial irradiation exposure in mice

[1$]Charles P. Hinzman, [2$]Janet E. Baulch, [3]Khyati Y. Mehta, [3]Michael Girgis, [3]Shivani Bansal, [3]Kirandeep Gill, [3]Yaoxiang Li, [2]Charles L. Limoli, *[1,3]Amrita K. Cheema

[1]*Department of Biochemistry, Molecular and Cellular Biology, Georgetown University, Georgetown University Medical Center, Washington D.C. 20057*

[2]*Department of Radiation Oncology, University of California, Irvine, CA 92697*

[3]Lombardi Comprehensive Cancer Center, *Department of Oncology, Georgetown University Medical Center, Washington D.C. 20057*

<u>Supplementary Figure S1</u>: Quantitative ELISA and nanoparticle tracking analysis (NTA) of EV samples. (A) Quantitative CD-63 ELISA counts for sham and irradiated EV samples 2 days (top) or 2 weeks (bottom) post-irradiation. **(B)** Mode diameter, in nanometers (nm) for EVs isolated at 2 weeks post-irradiation. *P*-values: * = ≤ 0.05, ns = not significant

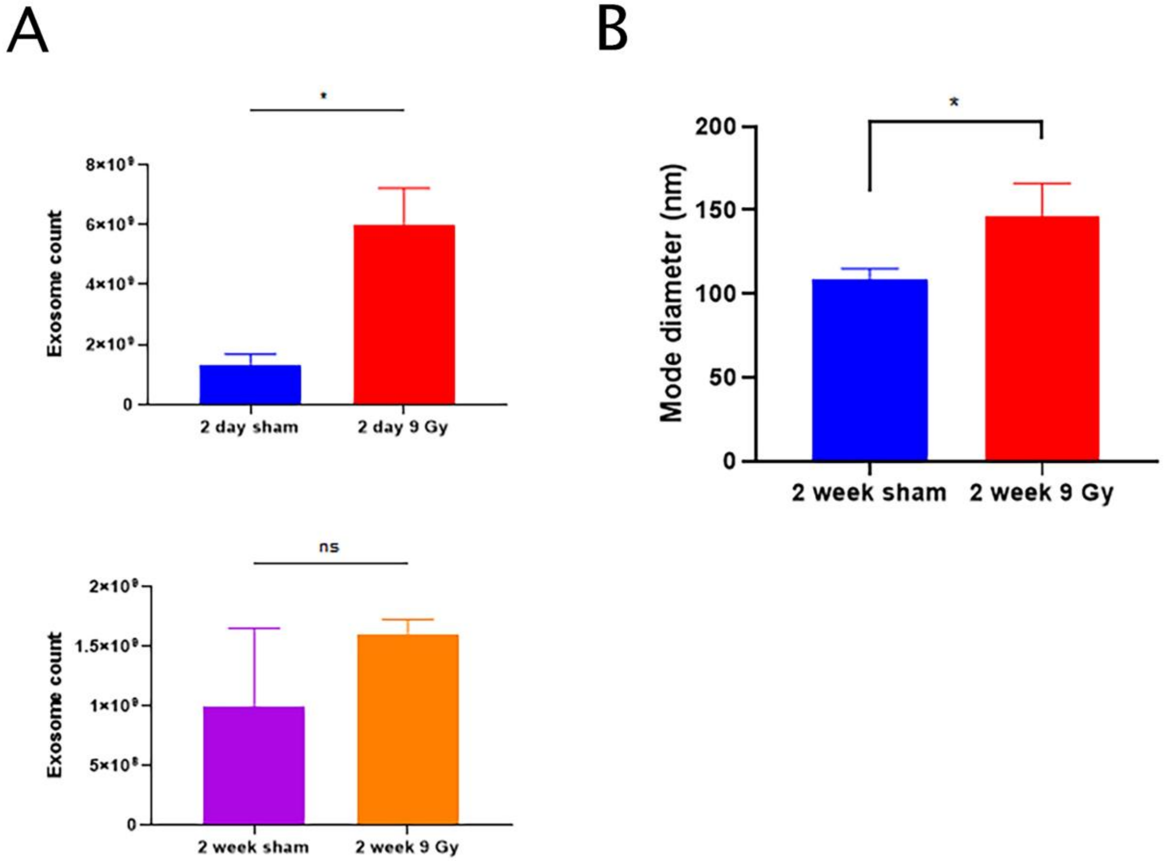

<u>Supplementary Figure S2:</u> EV biomarkers can accurately discriminate radiation exposure. A biomarker panel of triglyceride species was analyzed for predictive performance using a receiver operating characteristic (ROC) curve. A panel of just 2 triglyceride species, TG(14:0/16:0/20:2) and TG(16:0/18:1/18:2) was robust enough to accurately delineate between mice which received 9 Gy cranial irradiation and sham irradiated mice 2 days post-irradiation.

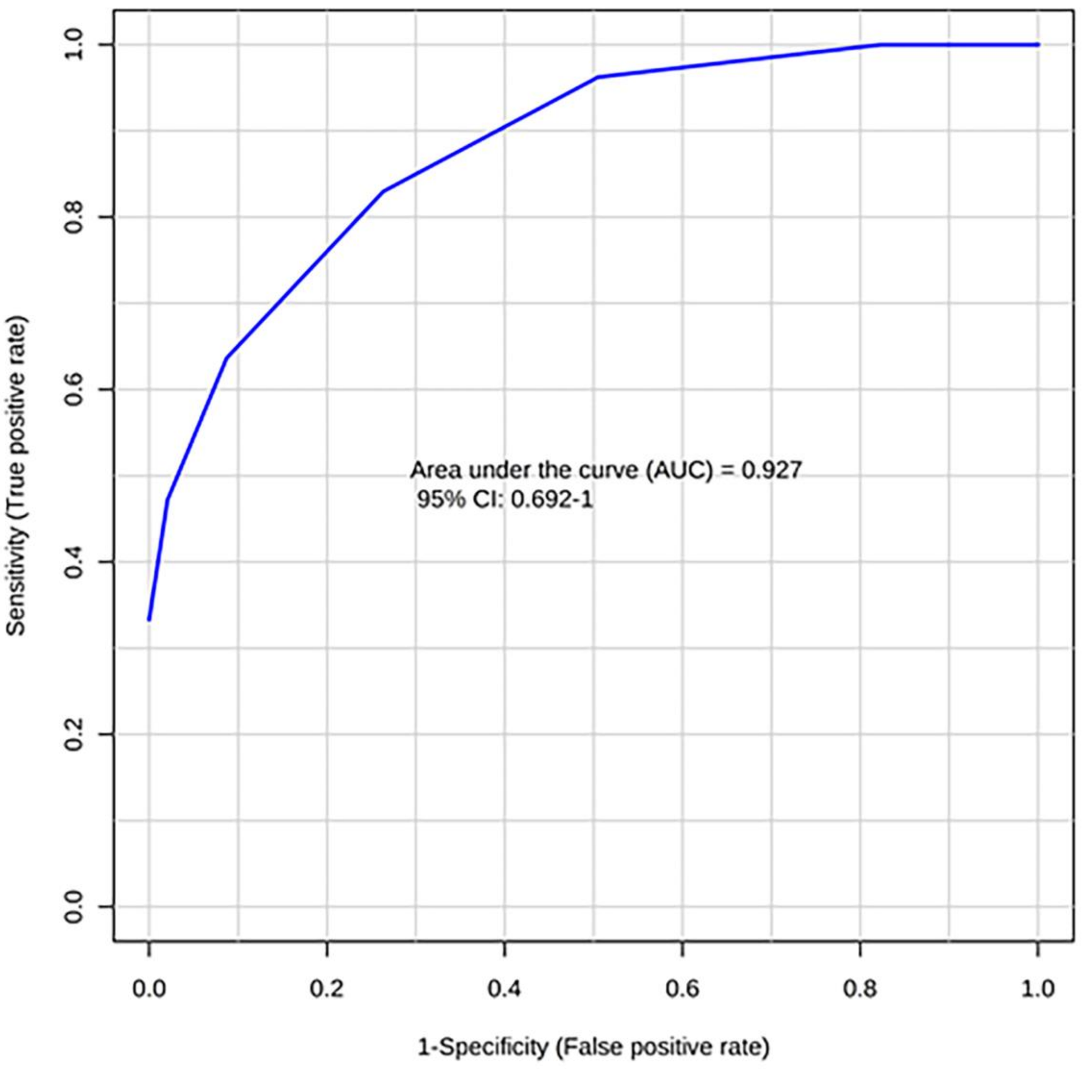

<u>Supplementary Figure S3:</u> Triglyceride (TG) species can predict radiation exposure from EVs, but not total plasma. (A) Receiver operating characteristic (ROC) curve demonstrating predictive ability of TG panel for cranial radiation exposure. **(B)** LC-MS/MS normalized response values for TG species in plasma from mice exposed to 9 Gy cranial irradiation.

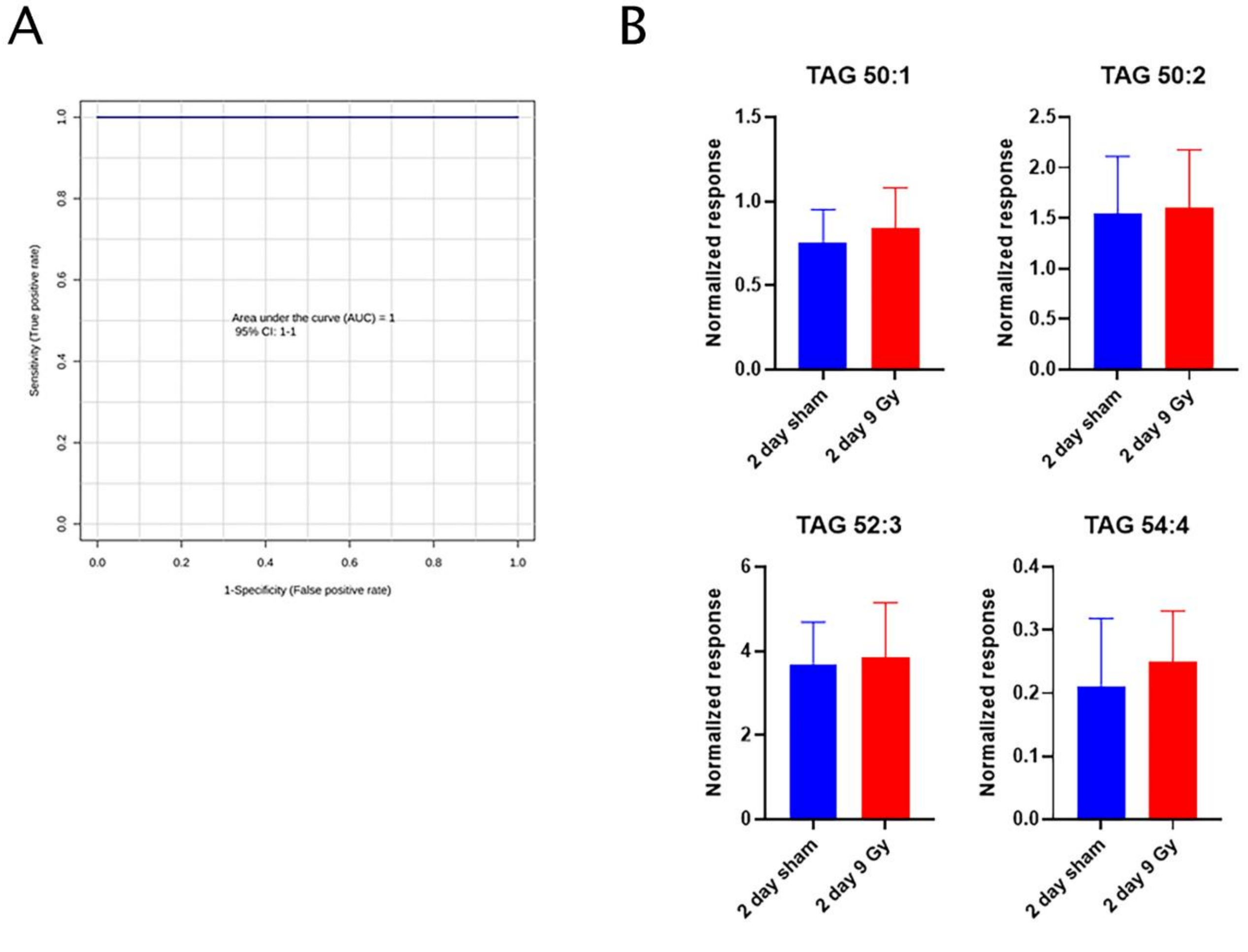

Supplement: Supplementary file 1 — Supplemental Figures S1-S3 [file 41598_2019_45970_MOESM1_ESM.pdf]
